# Evaluation of Land Use Regression Models for Nitrogen Dioxide and Benzene in Four US Cities

**DOI:** 10.1100/2012/865150

**Published:** 2012-11-25

**Authors:** Shaibal Mukerjee, Luther Smith, Lucas Neas, Gary Norris

**Affiliations:** ^1^National Exposure Research Laboratory, Office of Research and Development, U.S. Environmental Protection Agency, Mail Code E205-03, Research Triangle Park, NC 27711, USA; ^2^Alion Science and Technology Inc., Durham, NC 27713, USA; ^3^National Health and Environmental Effects Research Laboratory, Office of Research and Development, U.S. Environmental Protection Agency, Mail Code 58-A, Research Triangle Park, NC 27711, USA

## Abstract

Spatial analysis studies have included the application of land use regression models (LURs) for health and air quality assessments. Recent LUR studies have collected nitrogen dioxide (NO_2_) and volatile organic compounds (VOCs) using passive samplers at urban air monitoring networks in El Paso and Dallas, TX, Detroit, MI, and Cleveland, OH to assess spatial variability and source influences. LURs were successfully developed to estimate pollutant concentrations throughout the study areas. Comparisons of development and predictive capabilities of LURs from these four cities are presented to address this issue of uniform application of LURs across study areas. Traffic and other urban variables were important predictors in the LURs although city-specific influences (such as border crossings) were also important. In addition, transferability of variables or LURs from one city to another may be problematic due to intercity differences and data availability or comparability. Thus, developing common predictors in future LURs may be difficult.

## 1. Introduction

Compliance-oriented air pollution monitoring, even for population-oriented monitors, is generally conducted at only a few locations in a city to reflect higher population exposures. This provides limited information on spatial variability of urban air pollution [1]. LURs have been increasingly used in assessing intraurban gradients for population exposure assessments to support spatial-based air quality and epidemiological studies. LURs are GIS-statistical techniques used to estimate spatial distribution of air pollution concentration gradients in urban areas. In brief, LURs are multiple regression models with a basic functional form

(1) Y=b0+b1∗Traffic+  b2∗Population+b3∗Point Source⋯,

where *Y* denotes mean pollutant concentration and *b*_*i*_'s are predictor variable coefficients estimated by the procedure [2]. The generic variable groups (traffic, population, point source, and others) may have multiple variables associated with them (see Table 1). LURs can be linear, semiparametric, or based on the distribution of pollutant data versus predictor variables. LURs have progressed with increased use of passive air sampling and advances in portable samplers [2–4].

The US Environmental Protection Agency (EPA) has been involved in LUR studies in El Paso, Detroit, Dallas, and Cleveland (referred to here as the four cities) to support air quality and respiratory health studies [5, 6]. These LUR studies were conducted in El Paso and Detroit during multiweek campaigns during the winter and summer, respectively. Dallas and Cleveland studies were conducted during summer and winter seasons; however, in Dallas the seasons were separated by over a year. LUR results from these four cities are published elsewhere [7–10]. This paper discusses how their development and comparison can address LUR application across these and potentially other US cities.

## 2. Methods

### 2.1. Cities and Predictor Variables Used

El Paso and Dallas are in the US state of Texas. El Paso is on the western tip of Texas and sits between the Rio Grande River and the Franklin Mountains. The Rio Grande River is part of the US-Mexico border region; Ciudad Juárez, Mexico's fourth largest city, is adjacent to El Paso. Dallas, part of the Dallas-Fort Worth metroplex, is in north-central Texas and has flat terrain. Detroit, MI and Cleveland, OH are Great Lakes cities with heavy industry such as automobile and iron and steel production and have flat to gently-rolling terrain; Detroit is a US-Canada border city adjacent to Windsor, ON. As encountered for many urbanized areas, mobile sources are a major source of air pollution in the four cities.

LURs were constructed separately in the four cities. A GIS platform was used to develop predictor variables to be used in the regression analyses and to select monitoring sites. In the LURs, the general groups of variables were distance to roadways, traffic intensity, population density, land use, emissions levels, and city-specific variables such as distance to border crossings or distance to Lake Erie (Table 1). Traffic data were obtained from local county or metropolitan planning organizations, population figures were obtained from the latest US Census, and emissions were obtained from the EPA National Emissions Inventory. Other variables were obtained from ArcGIS (ESRI, Redlands, CA, USA) and related databases. Statistical analyses, including development of LURs, were implemented in SAS version 9.2 (SAS Institute, Cary, NC, USA).

A large number of GIS variables (typically > 40) were developed from the databases. For use in the LURs, potential explanatory variables were selected within their appropriate variable group to exhibit a reasonable amount of variability across the geographic study area and have low correlation with other potential predictors. To select the variables, separate correlation analyses for variable groups were conducted, and the correlations were examined between variables from different types of groups (e.g., population density and traffic intensity). Table 1 shows the groups of predictor variables employed in the LURs for each of the four cities.

**Figure 1:**
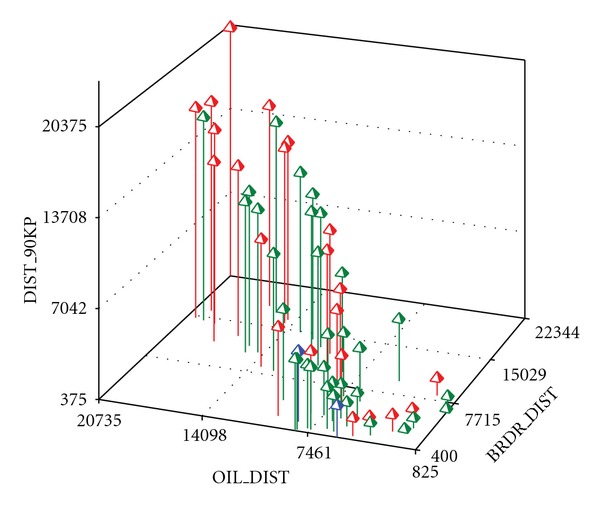
Example of El Paso school sites chosen (red) to be representative of all other school sites (green) for the variables of distance to petroleum facility point source (OIL_DIST, m), distance to nearest road segment ≥90,000 cars/day (DIST_90KP, m), and distance to nearest border crossing (BRDR_DIST). (Blue sites are compliance sites).

Variables chosen as potential predictors were also used to select monitoring locations. In the LURs, schools or fire stations were used to represent neighborhood-scale, ambient exposures. Such sites had secure, free air-flow sampling locations, and similar sampling heights of approximately 1.5–2 m. Sites were ranked on each potential predictor and ultimately selected based on their joint predictor variable ranges and variabilities. Chosen sites had similar correlation structure among potential predictors as the unmonitored sites. Cluster analysis was also used to ensure that the chosen sites adequately covered the mathematical space defined by the potential predictors. (The mathematical space is established by the variables' ranges and their overall correlation structure.) Figure 1 is an example from El Paso of how multiple variables were considered jointly in the site selection process. Note that the chosen school sites (in red) were representative of all possible school locations (in green) in El Paso in terms of the joint mathematical space spanned by the variables distance to the nearest petroleum facility point source, distance to the nearest border crossing, and distance to the nearest road segment ≥ 90,000 vehicles/day. The numbers of monitored sites for each city are presented in Table 2.

### 2.2. Passive Samplers

Passive sampling methods, which are typically employed in LUR studies since they are field portable and economical, were used. NO_2_ was sampled with Ogawa badges (Ogawa & Co., Pompano Beach, FL, USA). VOC samples were collected using 3 M OVM samplers in El Paso and PE tubes packed with Carbopack X sorbent (Supelco, Inc., Bellefonte, PA, USA) in Detroit and Dallas; no passive VOCs were collected in Cleveland. (Ammonia and passive aerosol sampling were conducted in Cleveland; see [10].) These samplers have been evaluated in these LUR studies and found to be comparable to Federal and other reference methods [9–12]. At least one compliance site operated by the local air pollution control agency in each city also had passive samplers to evaluate their accuracy with reference methods. Passive measurements at compliance sites were not used to develop LURs, but rather to evaluate LUR predictions.

Passive samplers were deployed for week-long sampling integrals to represent chronic exposures. During the given studies, passive samples were deployed concurrently at all sites. Monitoring time frames typically lasted five weeks during a season; however, sampling in El Paso lasted two weeks. Ambient monitoring was conducted in El Paso in November/December 1999, Detroit in summer 2005, Dallas in summer 2006 and winter 2008, and Cleveland in summer 2009 and winter 2010. All samplers were deployed concurrently during study periods and housed in appropriate shelters. Further details on the field sampling and lab analysis methods are presented elsewhere [9–12].

## 3. Results

### 3.1. Overall Levels

Summary statistics of air pollution data at monitoring sites from the four cities are shown in Table 2. NO_2_ and benzene (representing VOCs) concentrations were comparable across the cities, but median El Paso levels were the highest observed. Complex terrain conditions such as the central valley concentrating emissions from El Paso and Ciudad Juárez [13–15] may have been a factor in higher pollutant concentrations encountered there. For NO_2_, median levels were lowest in Cleveland during summer. This may have resulted from higher chemical reactivity in summer transforming NO_2_ into secondary products such as ozone. Another possibility may be that some industrial sources in Cleveland were shuttered or operating at reduced capacity during the summer monitoring, but activity increased during the winter. (Note that median levels were higher during winter in Cleveland than in summer.) The weekly passively monitored NO_2_ levels were below the annual EPA National Ambient Air Quality Standard of 53 ppb.

### 3.2. LUR Results

Based on visual inspection of plots of the air pollution data versus predictor variables and residual analyses, multiple linear regression models were used for LURs in Detroit, Dallas, and Cleveland, and semiparametric regressions (as generalized additive models) were applied in El Paso. Significant variables (5% level) and model predictive capacity (as *R* ^2^) are shown in Table 3 for the NO_2_ and benzene LURs from the four cities. Cleveland LUR results are shown for NO_2_. (Specific predictor variables and their coefficients in LURs are presented elsewhere in the models' results for El Paso [7], Detroit [8], Dallas [9], and Cleveland [10].) Though generally successful, the LURs yielded low *R* ^2^ values (<50%) for NO_2_ in both seasons and benzene in winter in Dallas and for benzene in Detroit. *R* ^2^ values were highest in El Paso and Cleveland. In El Paso, distinct gradients from complex terrain may have helped delineate spatial differences. The Cleveland LURs were able to benefit from the prior LUR study experiences which suggested a more refined approach to some of the predictor groups (such as total emissions within a buffer zone), the addition of new variable types (such as secondary and local road length), and the explicit incorporation of season.

As shown in Table 3, traffic influences and point source emissions were important predictor variables for LURs from the four cities. City-specific influences such as distance to an international border crossing were confirmed to be important for both border cities. Dominant sources such as traffic and industrial/other point sources were common for the four cities; reviews of LUR studies have confirmed these sources as common predictor variables [2, 4, 16]. However, local influences (such as border crossings) should also be considered when attempting to derive common exposure metrics from data collected in different cities.

Table 3 indicates both consistent and mixed responses to different predictor variables across the cities studied. For example, pollutant concentrations exhibited an (*a priori*) expected increase with traffic intensity and population density when these were found to be significant. However, both significant increases and decreases of pollutant levels were found with respect to the distance from high traffic volume and medium traffic volume roadways and distance to the nearest border crossing, depending upon the city and pollutant. Furthermore, Table 3 suggests differing behavior with respect to proximity to point sources.

These apparent inconsistencies may in part be due to characteristics of the local road networks within the cities, partly to the varying definition of the predictor variables between the cities, and seasonal effects. For example, in Detroit and Dallas, NO_2_ levels are influenced by distance to both medium and high traffic volume roads, but the effects are in opposite directions in the two cities. In Detroit, NO_2_ increases as distance to a high traffic road increases but decreases the farther from a medium traffic road; however, in Dallas, the roles of the roadways are reversed in summer. This may simply be a reflection of both the overall numbers of medium and high traffic roads as well as their relative locations in the two cities. Seasonal effects were an influencing factor in Cleveland LURs. Dallas may have indicated seasonal effects, but the seasonal data were from different years. El Paso and Detroit sampling was for only one season. Data collection in these from two seasons within the same year may have tempered the inconsistencies noted above.

Another factor contributing to the apparent inconsistencies in Table 3 may be related to the varying meaning of point source proximity from city to city. For example, in El Paso, the only type of point source considered (aside from a border crossing) was a petroleum facility, whereas in the other cities no restriction was made with respect to the type of facility. In addition, point source influence in Cleveland was expressed via emissions intensity which accounted for all facilities within a fixed radius buffer, whereas only the simple distance to a facility was used in other three prior cities. While this varying definition with respect to point sources allowed the subsequent LUR modeling to benefit from the lessons learned previously, it does complicate the interpretation when comparing results across the cities. 

**Figure 2:**
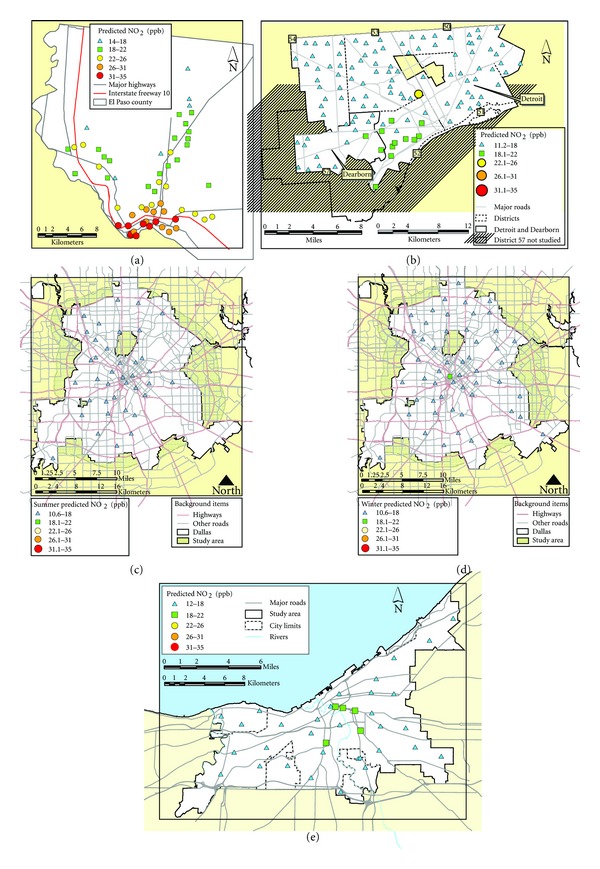
LUR predicted NO_2_ concentrations: (a) El Paso; (b) Detroit; (c) Dallas summer; (d) Dallas winter; (e) Cleveland (average of summer and winter). NO_2_ gradients are the same scale in all cities for comparison.

LUR modeling revealed spatial gradients for all pollutants. For example, NO_2_ was generally higher in downtown, industrial, central valley, and high traffic areas of cities where such emission activities would be located (Figure 2). Comparison of LUR predictions to passive measurements at compliance sites indicated general agreement given the generally low concentrations with percent differences of 0–33% for NO_2_ and 4–32% for benzene. Spatial differences were also noted for benzene which tended to be influenced both by traffic and point sources in the three cities where it was measured (see Table 3).

### 3.3. Evaluation of Common Variables

Transferability of LURs to different study areas has been suggested as a cost-effective alternative to developing new LURs; LURs transferred to similar types of cities have been evaluated with limited success [2, 17, 18]. Comparison of model power of transferred LURs versus locally developed LURs suggested variables from where monitoring was performed were preferred [17, 18].

To this end, we evaluated common variables considered for LURs in Detroit and Cleveland to determine whether LUR variables had similar values that could be transferable between the two cities. We did this comparison using these cities since they were geographically similar and had similar emission sources. NO_2_ was used with the variables to evaluate distribution. Summer data from Cleveland were compared with Detroit measurements that were also collected during summer.

**Figure 3:**
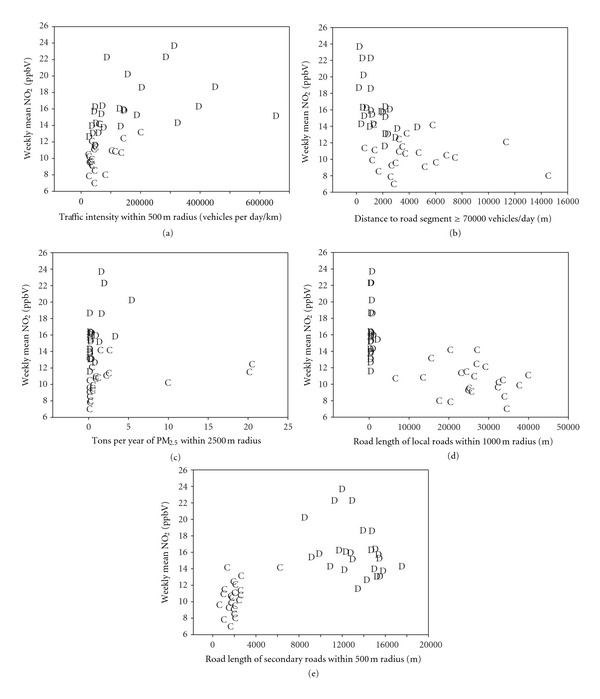
NO_2_ concentration using common variables in Detroit (D) and Cleveland (C).

Figures 3(a)–3(e) display scatterplots for NO_2_ using common variables in Detroit (D) and Cleveland (C). Variables such as traffic intensity within 500 m radius (Figure 3(a)), distance to road segment with a traffic volume of at least 70,000 vehicles per day (Figure 3(b)), and PM_2.5_ emission sources as tons per year within 2500 m radius (Figure 3(c)) showed similar distributions between the two cities. However, dichotomous relationships between cities were seen for road length variables for local roads within 1000 m (Figure 3(d)) and secondary roads within 500 m (Figure 3(e)). Figure 3(d) reveals a large number of zeros for local roads in Detroit; most roads were not designated as “local” near Detroit sites. Road length variables were calculated from ArcGIS databases so the potential for misclassification with these variables is based on classification codes internal to ArcGIS databases. (Road length variables were not applied in the Detroit LURs.)

## 4. Discussion and Conclusions

LURs were successfully developed from passive sampling networks in the four cities. Considered conjointly, the studies confirm flexibility and universality of traffic and other urban source variables in LURs for predicting air pollutant concentrations. As with the measured pollutants, predictor variables should be collected from the local study area for reliable spatial predictions.

Gaseous air pollutants were generally similar across the cities, but higher levels in El Paso may have been due to complex terrain concentrating pollutants from El Paso and Ciudad Juárez. Traffic, point source, and population counts were important predictors in the LURs despite major differences in geographic characteristics between the four cities. These variable groups were similar to those used in other LURs [4, 16]. Variables calculated from such data should be considered as potential predictors when developing candidate variables for LURs in other cities as well as developing common exposure metrics. However, city-specific influences (such as border crossings and elevation) can also be important. The potential misclassification of GIS data such as primary, secondary, and local roads can result in variable differences between cities that can adversely affect commonality with other areas. In addition, results from Dallas and Cleveland suggest that season can also play a role in predicting pollutant concentrations [9, 10].

LURs were developed during their respective monitoring periods, and prior experience was used to inform the subsequent efforts. For example, traffic variables in El Paso, Detroit and Dallas used distance to roads carrying various vehicle counts and traffic intensity. In Cleveland, categories of local and secondary road lengths within various buffers were added to better capture the potential total impact of traffic. Point source emission variables in El Paso, Detroit, and Dallas included the distance from the nearest large emitters of a given pollutant; this was revised for Cleveland by using emissions densities within various buffer sizes, thus incorporating all available emissions information.

The potential of differential seasonal impacts was explored in Dallas, though unfortunately the relatively large gap between the actual field monitoring periods precluded a definitive conclusion regarding potential seasonal effects. In Cleveland, however, season was explicitly used as a predictor itself and as an interacting factor with other predictors. El Paso and Detroit LURs could not use season as a predictor since data were only measured during winter and summer, respectively.

Finally, common types of predictor variables can be applicable in LURs from city to city. However, coefficients in LUR models can be significant or not, and even common significant predictor variables (e.g., distance to nearest road) can have opposite effects depending on city-to-city differences in source and pollutant measures. In addition, transferability of variables or LURs from one city to another may be problematic due to differences in how GIS data are defined. Differences in roadway characteristics may not be incorporated into the definition of the predictor variables. For example, considerations such as elevated and depressed roadways, tunnels, or overpasses were not considered in defining the GIS variables used in these four cities. In addition, it was noted that the definition of local and secondary road categories was different between Detroit and Cleveland, despite their geographic and emission similarities. Though extracted from the same standard ArcGIS databases routinely used to develop road network variables for LURs, it was apparent that different criteria had been used to categorize roads as local or secondary in the two cities. Inherent misclassification of roads could only be rectified by transportation surveys. Caution should be exercised when evaluating similarities or differences of such variables from city to city. Another complicating factor for the transferability question is the importance of city-specific factors; for example, El Paso and Detroit have border crossings unlike Dallas and Cleveland.

In conclusion, neighborhood-scale spatial gradients were encountered in the pollutants confirming the influence of traffic and other urban influences. Traffic and other urban variables were important predictors in the LURs although city-specific influences and season of the year may also be important. However, transferability of specific variables or LUR predictive equations from one city to another may be problematic due to intercity differences and data availability or comparability. Thus developing common predictors in future LURs may be difficult.

## Figures and Tables

**Table 1:** Group types for potential predictor variables^a^.

Predictor variable groups and subgroups from GIS	El Paso	Detroit	Dallas	Cleveland
(1) Traffic				
Distance to nearest low-traffic road (m)^b^	X^c^			
Distance to nearest medium-traffic road (m)		X^d^		X^e^
Distance to nearest high-traffic road (m)	X^f^	X^f^	X^g^	X^h^
Traffic intensity within set buffers (vehicles per day/km)	X	X	X	X
Length of local roads within set buffers (m)				X
Length of secondary roads within set buffers (m)				X
(2) Area and point				
Open area within set radii (km^2^)				X
Population density within census block group or set radii	X	X	X	X
Point source emitters (categorical or continuous)	X^i^	X^i^	X^i^	X^j^
(3) City specific				
Elevation (m)	X			
Distance to nearest international border crossing (m)	X	X		
Distance to airport (km)				X
Distance to lake (km)				X
(4) Season			X	X

^a^Specific variables and their sources are detailed elsewhere for El Paso [7], Detroit [8], Dallas [9], and Cleveland [10]. ^b^Units in parentheses. ^c^road > 10,000 vehicles/day. ^d^  ≥50,000 vehicles per day. ^e^  ≥40,000 vehicles/day. ^f^  ≥90,000 vehicles/day. ^g^  ≥140,000 vehicles/day. ^h^  ≥70,000 vehicles/day. ^i^Distance (m) from emission sources. ^j^Emission sources within set buffers.

**Table 2:** Median pollutant concentrations (all above method detection limits) in the four cities^a^.

Pollutant	El Paso (22 schools)	Detroit (25 schools)	Dallas (24 fire stations)	Cleveland (22 fire stations)
NO_2_	22 (11, 37)	16 (11, 24)	12 (4, 25)^b^	10 (2, 29)^d^
			14 (2, 22)^c^	18 (0, 25)^e^
Benzene	777 (489, 1531)	466 (338, 698)	232 (83, 388)^b^	Not measured
			357 (247, 538)^c^	

^a^Medians calculated over all sites and weeks. Units for NO_2 _in ppb; benzene in ppt. Minimum and maximum values in parentheses.

^b^Summer 2006.

^c^Winter 2008.

^d^Summer 2009.

^e^Winter 2010.

**Table 3:** Model *R* ^2^ and significant variables (5% level) in NO_2_ and benzene LURs.

Model *R* ^2^ (%)	El Paso		Detroit		Dallas		Cleveland
	NO_2_	benzene	NO_2_	benzene	NO_2_	benzene	NO_2_
	97	93	82	43	34^a^/48^b^	72^a^/49^b^	96
Distance to nearest low traffic road							
Distance to nearest medium traffic road			*▼* ^ c^	*▼*	▲/		▲
Distance to nearest high traffic road			▲^d^		*▼*/	*▼*/	
Traffic intensity within set buffers	▲				▲/▲	▲/	▲
Length of local roads within set buffers							*▼*
Length of secondary roads within set buffers							*▼*
Open area within set radii							*▼*
Population density within census block group or set radii	▲	▲		▲			
Point source (categorical or continuous)	*▼* and ▲^e^	*▼* and ▲	*▼*	*▼*	*▼*/*▼*		♦^f^
Elevation							
Distance to nearest international border crossing	*▼*	*▼*	*▼*	▲			
Season							♦
Seasonal interaction of point source and population density categories							♦

^a^Summer.

^b^Winter.

^c^Significant (5% level) decrease.

^d^Significant (5% level) increase.

^e^Decrease followed by increase.

^f^Categorical variables (significant 5% level).
